# A blockchain-based information market to incentivise cooperation in swarms of self-interested robots

**DOI:** 10.1038/s41598-023-46238-1

**Published:** 2023-11-21

**Authors:** Ludéric Van Calck, Alexandre Pacheco, Volker Strobel, Marco Dorigo, Andreagiovanni Reina

**Affiliations:** Institut de Recherches Interdisciplinaires et de Développements en Intelligence Artificielle (IRIDIA), Université Libre de Bruxelles, Brussels, Belgium

**Keywords:** Mathematics and computing, Information technology

## Abstract

Robot swarms are generally considered to be composed of cooperative agents that, despite their limited individual capabilities, can perform difficult tasks by working together. However, in open swarms, where different robots can be added to the swarm by different parties with potentially competing interests, cooperation is but one of many strategies. We envision an information market where robots can buy and sell information through transactions stored on a distributed blockchain, and where cooperation is encouraged by the economy itself. As a proof of concept, we study a classical foraging task, where exchanging information with other robots is paramount to accomplish the task efficiently. We illustrate that even a single robot that lies to others—a so-called Byzantine robot—can heavily disrupt the swarm. Hence, we devise two protection mechanisms. Through an individual-level protection mechanism, robots are more sceptical about others’ information and can detect and discard Byzantine information, at the cost of lower efficiency. Through a systemic protection mechanism based on economic rules regulating robot interactions, robots that sell honest information acquire over time more wealth than Byzantines selling false information. Our simulations show that a well-designed robot economy penalises misinformation spreading and protects the swarm from Byzantine behaviour. We believe economics-inspired swarm robotics is a promising research direction that exploits the timely opportunity for decentralised economies offered by blockchain technology.

## Introduction

Drawing inspiration from collective behaviours observed in natural systems—from the choreographic dances in the sky by flocks of birds to the laborious societies of social insects—swarm robotics deals with the design of decentralised systems where large groups of robots can collectively perform a task more efficiently than when operating as individuals^1,2^. Robot swarms operate without any centralised control; rather, the swarm coordinates through self-organisation among its constituent units, the robots, that can only exploit local environmental sensing and limited communication and actuation capabilities. This decentralised architecture has the potential to enable parallel execution of the task, scalability of performance with increasing swarm size, and fault tolerance to individual malfunctioning. While these advantages are the key motivation to swarm robotics, they are not automatically present in every decentralised system; rather, they can only be attained through careful design^3–5^. Indeed, thanks to their high redundancy, robot swarms are fault tolerant against individual failures that do not influence other robots’ behaviour. However, in the presence of Byzantine robots, i.e., faulty or malicious robots that are capable of disrupting others’ behaviour, the swarm may not be able to perform the expected task. Our study shows the fragility of traditional swarm robotics studies when Byzantine robots are considered and how robustness to Byzantine robots can be enabled through economics-inspired design.

While most research in swarm robotics focused on coordination among fully cooperative individuals, relatively limited research has investigated how to make robot swarms resilient against Byzantine robots^2^. Most effort has been dedicated to solutions to secure collective decision making, where the swarm is tasked with making a consensus decision on the best option available in the environment (e.g. an environmental feature or a site where to aggregate)^4,6–8^. As such, Byzantine strategies consist of trying to delay consensus (possibly indefinitely), or make the swarm choose a sub-optimal option. A recurrent type of Byzantine behaviour disrupting best-of-*n* problems is the zealot (or stubborn) behaviour, which is the behaviour presented by a robot that ignores social information and is immovable in its opinion. Research has investigated what are the best rules for updating the robot’s opinion in order to mitigate the impact of such behaviours, without using any mechanism to identify malicious or malfunctioning agents^6,7,9^.

Previous work has also investigated how robots can protect the collective efficiency of the swarm through either individual or systemic protection mechanisms. Mechanisms of individual protection consist of letting the robots use opinion update rules designed to mitigate the impact and spreading of misinformation, e.g. through methods of outlier detection^8,10–12^. A different idea consists of enforcing systemic rules, independent of the robot’s opinion update rules, that protect the swarm from Byzantines’ misinformation. Systemic protection has only recently been considered a viable solution for swarm robotics, thanks to the development of distributed ledger technology. Blockchains and smart contracts enable the distributed execution of tamper-proof algorithms unlocking new possibilities for controlling and securing robot swarm behaviour^13^. For example, Strobel et al.^3,4,14,15^ have shown how blockchain-secured robots can prevent Byzantines from harming collective environmental monitoring, and Pacheco et al.^16^ have showcased the possibility of regulating in real time the collective behaviour of a foraging robot swarm.

In this study, we explore the idea of employing economic incentives for encouraging cooperation among robots, and most importantly to penalise the spreading of harmful misinformation. By exploiting the timely advent of blockchain technology, we suggest a paradigm shift in the design of collective behaviour for robot swarms, which have been traditionally inspired by the innate cooperative behaviour of eusocial insects^2^. In contrast, we do not assume cooperative behaviour by every robot; rather, the coordination of self-interested individuals is the consequence of well-designed economic rules. Thus, we believe that economics-inspired swarm robotics can sprout from recent successful application of similar economic mechanisms in the context of distributed ledger technology (e.g. DeFi and DApps). In line with previous studies^3,4,17^, we assume that the blockchain is maintained by the robots and that each robot is a blockchain node in the decentralised network. Robots exchange information through transactions on the blockchain, and they can agree on system-wide rules which are programmed in blockchain smart contracts: decentralised and tamper-proof algorithms that run on the blockchain^18,19^. Although tested in simulation, the models we employed for both simulated robots and simulated blockchain consider the relevant aspects of an implementation on a real blockchain-based robot swarm, as further discussed in detail in the *Methods* section. Additionally, the simulator, that we release as open source code, is structured in a way that offers easy extension to more sophisticated market rules and minimal programming overhead in order to be as accessible as possible to economics scholars for future extension and fostering interdisciplinary research.

### Social navigation in foraging robot swarms

Our goal is to show how decentralised economic incentives are a viable mechanism to enable security in swarms of robots that operate as a decentralised network and rely on local and partial knowledge. To do so, we consider collective foraging, a common swarm robotics benchmark application where robots are tasked with exploring the environment in order to locate, collect, and transport resources to given target locations^20–23^. Providing secure and efficient solutions to decentralised collective foraging can enable the deployment of robot swarms in several application scenarios, such as agriculture^24^, construction^25^, garbage collection^26^, and search and rescue^27^.

Foraging can be studied in various forms depending, for example, on how resources are scattered in the environment or the number of target locations^28^, and foraging studies can focus on various different aspects, for example, on the coordinated navigation of the environment^29,30^, on the collective transport of resources^31,32^, or on the allocation of robots between exploratory and exploitative tasks^33^. Here, we investigate central place foraging, where robots have to transport resources (also named *food*) to a single central depot location (named *nest*)^34^. We focus on a key aspect of collective foraging: *coordinated navigation* between food and nest locations without any global positioning system (GPS).

Studies about social navigation in swarms of foraging robots did not only borrow the terminology from biology but also adopted solutions and models from collective animal behaviour in order to engineer artificial swarms^35^. To efficiently navigate between locations without a GPS, previous studies investigated the use of different forms of coordination, which relied on either indirect (stigmergic) or direct communication.

Stigmergic communication is used by numerous ant species to recruit foragers to a profitable food source. Ants returning to the nest from a food source leave a pheromone trail that other ants can detect and tend to follow. Using this mechanism, ants are able to solve different types of problems, for example, choosing the best food source in the area^36,37^, or selecting the shortest path connecting food and nest^38^. Drawing inspiration from this method of indirect communication, stigmergy has also been employed to coordinate the navigation of foraging robot swarms. However, the artificial replacement of chemical pheromones can be challenging and only limited work succeeded, e.g. via ethanol^39^ or photochromic material^40^. Therefore, several previous studies relied on smart environments, e.g. using radio-frequency identification technology (RFID)^41^, augmented reality^30^, or specialised hardware^42^. Although these studies provided useful scientific insight, their application remains confined within research labs.

While simple living organisms relied on stigmergic communication for coordination, artificial swarms can also relatively easily exchange direct messages with structured content, potentially simplifying their deployment in the real world. Through direct local communication, robot swarms can coordinate and exploit a form of social odometry in order to efficiently navigate through the environment without GPS^29,43–45^. The robots, through what is often described as the “many-wrongs principle”^46,47^, compensate for individual odometry errors which are filtered out by mechanisms of information pooling and achieve higher navigation accuracy as a group than they would do as single individuals.

Several works made use of static chains of robots that acts as beacons to guide other mobile robots efficiently between the food and the nest^48–51^; however, this approach may suffer from low efficiency as the beacons are robots (potentially numerous in large environments) that do not actively contribute to the transportation task and may even restrict the movement of other robots. We base our work on an alternative solution that mitigates these drawbacks through the use of dynamic robot chains in which all robots in the chain move between food and nest sites, both transporting resources and guiding other robots^29,43–45^. In particular, Ducatelle et al.^44,52^ showed that robots, by sharing information about the last time they encountered a location and the relative positions between robots, were able to form an efficient dynamic chain that moved on the shortest path connecting food and nest, also in environments with obstacles. In our study, we extend this collective behaviour for social navigation, show that it is highly susceptible to false information, and propose new ways to deal with Byzantine robots.

## Results

**Figure 1:**
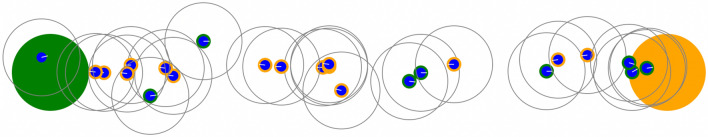
We simulate a swarm of 25 robots (blue circles) that move between food and nest sites (green and yellow circles). Robots filter out odometry noise and improve navigation efficiency implementing a social odometry algorithm based on local exchange of messages (the grey circles show the communication range). Through social odometry, the robots form a dynamic chain around the shortest patch connecting food and nest. The robots’ outline is the colour of their last visited site and the white line indicates the robot’s motion orientation. The simulator is easily extendable, open source, and available at https://github.com/ludericv/information-market.

We run multiagent simulations comprising 25 simulated robots—each with a different random level of odometry noise—that move between two sites, nest and food (Fig. 1). Our analysis keeps the navigation problem as simple as possible by letting robots move in an obstacle-free 2D environment where the shortest path between nest and food is a straight line. When a robot visits a site, it stores a noise-free ground truth on the site’s location, and, after every movement, through odometry it updates its path information, which consists of a 2D vector pointing to the site in the robot’s local coordinate system. Each robot stores two vectors, one for each site. Due to odometry noise, the vectors deviate from the ground truth over time, and without help a single robot quickly gets lost and is not able to reach the site. When a robot fails to reach the site because its path information is unreliable—i.e. it arrives at the location pointed by the vector without finding the site—it starts exploring the environment through a random walk.

To improve collective efficiency, we implement a social odometry algorithm^29,43–45,53^, through which robots exchange and combine each other’s path information with the objective of decreasing odometry noise and improving travel efficiency (i.e. follow a straight path and avoid random exploration). Robots store along with each vector an *age* attribute which is increased by one at every movement and conveys information about how many odometry updates have been applied to the vector. Through social odometry, a robot shares vectors and age with nearby robots, and when it receives a vector with an age lower than the one of its own vector (hence supposedly more accurate), the received vector is merged with the previous one (through age-weighted averaging)^29^. Using this strategy, the swarm in a relatively short time forms a coordinated chain of robots moving between the two sites, see Fig. 1.

**Figure 2:**
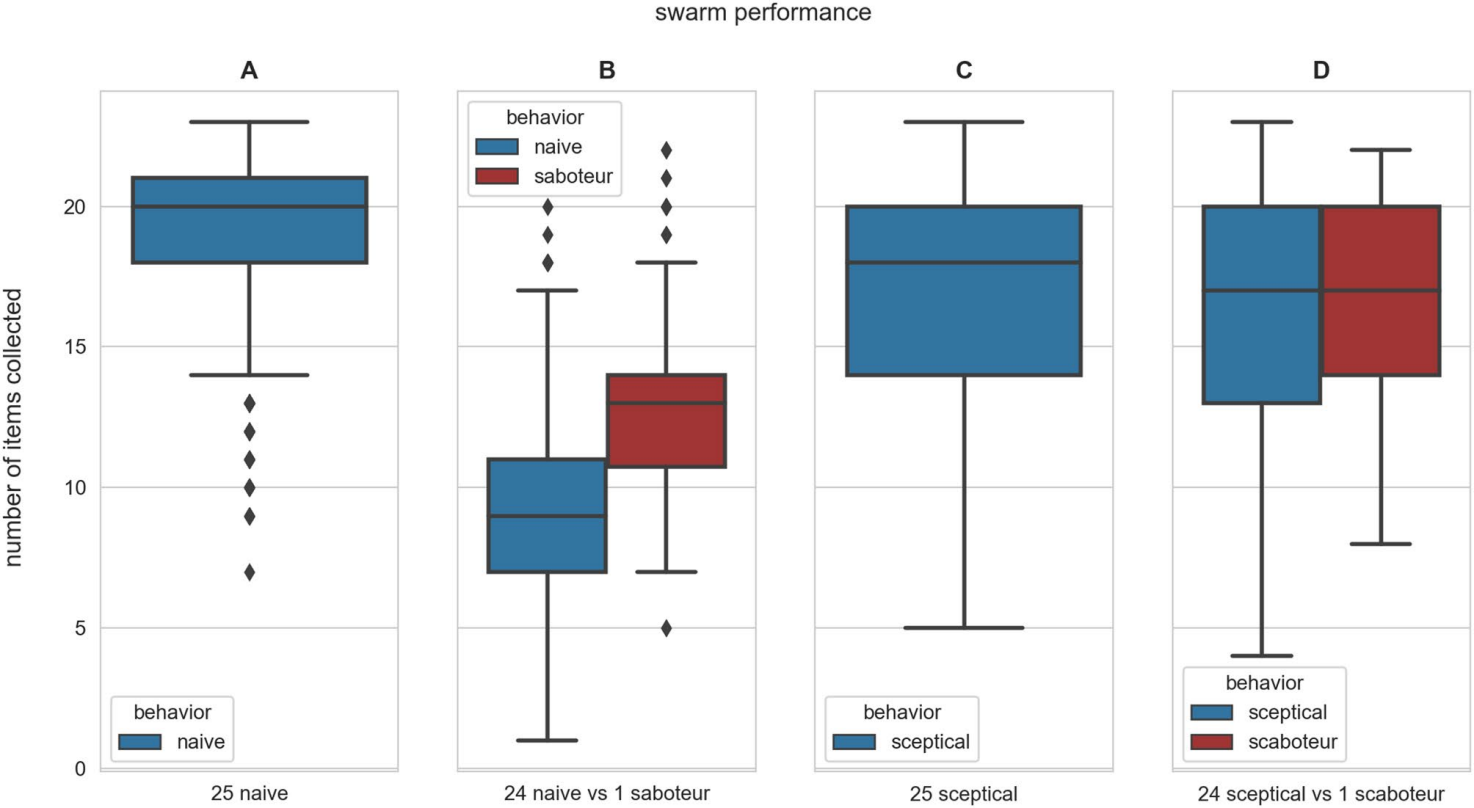
Box plots of the total number of items collected by each robot after 15 000 timesteps in 128 simulations per condition. Swarm size is kept constant at 25 robots in all our experiments, and we varied the swarm composition (indicated under each panel). The blue and red boxes show the results for honest and Byzantine robots, respectively. The boxes indicate the range between the data distribution’s first and third quartiles, the horizontal lines show the median, the whiskers extend in both directions to the last datapoint up to 1.5 IQR, and finally, the outliers are marked black diamonds. (**A**–**B**) Naive robot’s performance has a big decrease when a single saboteur is present. (**C**–**D**) Sceptical robots are instead more resilient to the presence of a saboteur.

Figure 2A shows that in an experiment lasting 15 000 timesteps, the median number of round trips that a robot completes is 20, that we measure as number of items collected at the food site and deposited at nest site. The distribution is negatively skewed (longer tail towards fewer collected items), showing that inaccurate robots (with high odometry noise) can fail to remain in the robot chain and drift away, collecting fewer items, but there is no considerable difference in performance among the upper half of the group (moderate and low noise) thanks to frequent updates and corrections of their information from other robots.

### Naive robot swarms are vulnerable to misinformation

Swarms composed of robots without any protection mechanism, which we name as *naive* robots, suffer a drastic drop in performance when a single Byzantine robot is included in the swarm. We run experiments with 24 *honest* naive robots (i.e. robots sharing their best estimate of the path) and one *saboteur*, a Byzantine naive robot that shares incorrect path information by sending vectors rotated by 90 degrees (because of malicious intention or a malfunctioning). While the saboteur shares incorrect path information, it stores the correct information which it uses to move between the sites. Movie S1 in the *Supplementary Information* shows that a single saboteur can have a dramatic impact on the swarm dynamics by systematically breaking the robot chain and sending robots in the wrong direction. Figure 2B shows that the number of items collected by the 24 honest naive robots (blue distribution) halves when one saboteur is present. Additionally, sharing fraudulent information (red distribution) offers the advantage of leading to a performance on average superior to the rest of the swarm.

Deploying a system that can be jeopardised by a single misbehaving robot is unacceptable. In order to improve the resiliency of the robot swarm, we introduce mechanisms to apply protection at the level of the single robot (individual protection) and of the swarm (systemic protection).

### Individual protection via sceptical robots

To counter the saboteurs, robots can individually adopt a more sceptical approach to what information they decide to use. *Sceptical* robots attempt to filter out fraudulent information by only using new information that is confirmed by a second piece of information. Both honest and Byzantine robots can implement this resiliency mechanism by which they average a new vector only when it is similar to the receiver’s navigation vector or when two distinct robots send similar vectors. We name honest sceptical robots as *scepticals* and saboteur sceptical robots as *scaboteurs*. The sceptical mechanisms is also implemented in saboteur robots in order to avoid that when there is more than one saboteur in the swarm, they would behave naively and sabotage each other more heavily than they sabotage honest robots.

Security comes with an efficiency cost. Increased scepticism in using others’ information delays correction of odometry errors and reduces individual and collective efficiency. Figure 2C shows that a swarm composed of 25 scepticals (without scaboteurs) has a lower performance than the naive counterpart (shown in Fig. 2A). The interquartile range is also wider than in the case of naive robots indicating that more robots can occasionally drift away from the robot chain; in particular, robots with high odometry noise can quickly accumulate large errors and move away from the chain when they do not immediately use the received information but wait for a second confirmation. Despite the cost, such a security mechanism grants the swarm resiliency against Byzantine robots. Figure 2D shows that a swarm composed of 24 scepticals and one scaboteur can maintain the same high performance level of Fig. 2C, which is significantly higher (almost the double) than the naive swarm under attack, in Fig. 2B. Furthermore, the performance of Byzantines is equivalent to the honest subgroup, rather than superior as for naive robots in Fig. 2B, hence making Byzantine behaviour less advantageous. Finally, we note that in sceptical swarms, Byzantine robots have a higher performance than in naive swarms (red distributions of Fig. 2B vs Fig. 2D) because the robot chain is safeguarded and all robots exploit it for efficient navigation.

### Systemic protection via blockchain-based smart contracts

This study proposes a different method to protect the swarm from harmful misinformation, an information market. The market’s economic rules can be enforced on a decentralised robot swarm through blockchain-based smart contracts, designed to reward honest behaviour and penalise misinformation spreading. In Section *How to transfer our solution to a blockchain-based smart contract*, we discuss in detail how the economic rules can be implemented in the blockchain smart contracts; here, we focus on market design^54^.

Robots receive a reward for every item they deposit in the nest, or, in other words, the nest becomes a marketplace where the robots sell items they collected at the food site and transported to the nest. Robots aim to maximise their reward as it can be a measure of their efficiency, as well as a monetary incentive for who deployed the robot or for the robot itself, which may seek economic independence^55^. Economic mechanisms can also regulate the exchange of information among self-interested robots, which aim to maximise their own profit, or accumulate higher wealth than others. Such self-interested robots may be reluctant in helping each other as they aim to prevail over other robots. For example, robots from different producers and with different owners can be part of an *open swarm*^4,13^, where robots join and leave freely at any time and work towards a common task. In such types of systems, robots only share useful information (e.g. direction to a site) in exchange of monetary recompense, that is, robots sell and buy information. This study shows that well-designed payment schemes can favour cooperation and coordination of swarms composed of self-interested robots. Among the tested payment schemes, we present the two most effective in penalising saboteur’s misinformation, both based on the *reward sharing* mechanism. Through reward sharing, the information-seller receives a share of the buyer’s reward once the latter deposits the next item. Therefore the seller does not have any immediate payment, instead the transaction is stored in the blockchain and the smart contract later distributes the reward among all sellers and the robot that transported the item.

#### Outlier penalisation

The *outlier penalisation* payment scheme is based on the assumption that Byzantine robots (in our case saboteurs) are a minority in the swarm and it aims to reduce the value of information that is categorised as an outlier—significantly different from other information. Because robots do not pay for information immediately when acquiring it but only once they have completed a round-trip (from nest to food, and back to nest), the smart contract is able to compare all of the information the robot gathered during the last round-trip and detect possible outliers. More specifically, once an item is deposited, the smart contract splits the reward into two equally sized shares, 50% of the reward goes to the robot that transported the item and the remaining 50% is distributed among all sellers of the last round-trip. The blockchain stores all the information exchanges as transactions, and the smart contract distributes reward to each transaction’s seller in a quantity proportional to the number of similar transactions. That is, a transaction with a high number of similar transactions receives a reward higher than a transaction with fewer similar transactions. Two transactions are considered as similar when the path information (i.e. the vectors to a site) have a difference in angle smaller than a threshold that in our experiments we set to $\Theta =30^\circ$.

We analyse how the outlier penalisation payment scheme distributes wealth among the robots. Robots increase their wealth by both depositing items at the nest site and by selling information to other robots. As the reward’s value can be chosen arbitrarily, the numeric value of a robot’s total wealth does not carry much meaning on its own, therefore we report the proportion of each robot’s wealth compared to the whole population’s wealth. Analysing the wealth proportion, we can compare different combinations of payment systems and reward mechanisms, and understand which robots get wealthier over time. In our experiments with 25 robots, when wealth is shared equally among all robots, each will have a $\frac{1}{25} = 4\%$ of the wealth; any deviation from $4\%$ indicates the presence of “richer and poorer” robots.

**Figure 3:**
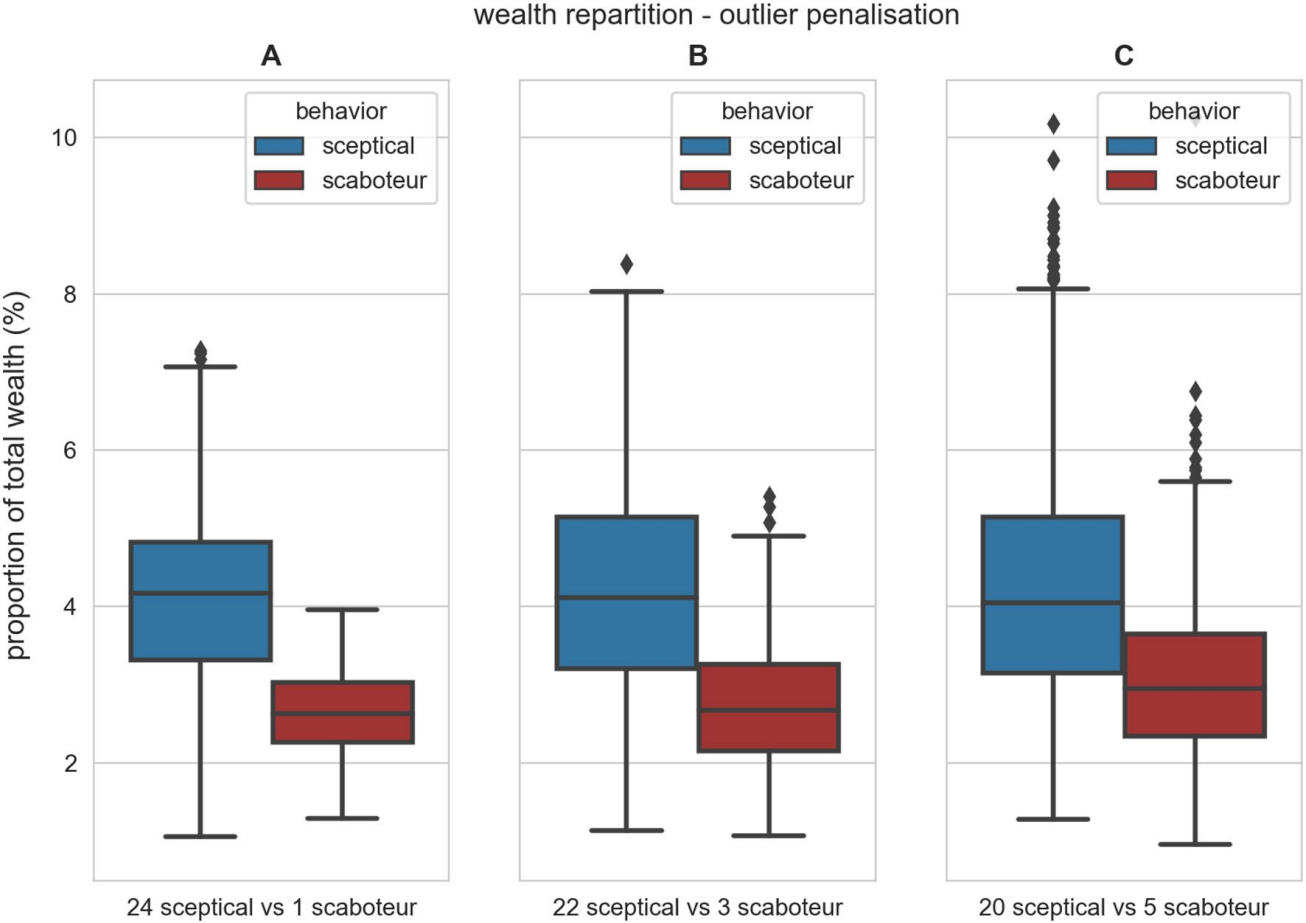
Box plots of the proportion of wealth of each robot after 15 000 timesteps in experiments with the outlier penalisation payment scheme (128 simulations per condition). We varied the swarm composition (indicated under each panel) in swarms composed of 25 robots. The blue and red boxes show the results for honest and Byzantine robots, respectively (see full description of the box plots in the caption of Fig. 2). In all tested conditions, the blue distribution is significantly higher than the red one (Mann-Whitney *U* test, *p*-value < 0.001 in all cases). The outlier penalisation payment scheme penalises more heavily the wealth of Byzantine robots when they are few, however the difference between the blue and red distribution decreases as the number of Byzantine robots increases.

Figure 3 shows how the outlier penalisation payment scheme distributes wealth in swarms with different numbers of scaboteurs. When there is only a single scaboteur in the swarm, the median of its wealth proportion is about $3\%$, while for honest sceptical robots it is slightly above 4%. As the number of saboteurs increases, the difference between the wealth distributions becomes less pronounced, yet they remain statistically different.

#### Outlier penalisation with staking

Even though the outlier penalisation payment scheme successfully penalises Byzantine robots, which receive a smaller share of reward than honest sceptical robots, the difference is minimal when more than one Byzantine is present in the swarm (Fig. 3). Additionally, Fig. S1 (in the *Supplementary Information*) shows that Byzantine robots increase their wealth throughout the experiment despite disseminating information classified as outlier (hence potentially fraudulent). Therefore, we design another payment scheme—*outlier penalisation with staking*—aimed at actively decreasing wealth of Byzantine robots. This payment scheme is based on the previous outlier penalisation scheme and has the additional mechanism of staking. Robots that sell path information must stake a fixed monetary amount (in our experiments 0.04 crypto tokens) in order to include their transaction in the blockchain. Staking consists of blocking crypto tokens that are unavailable to both the buyer and seller, and are released by the smart contract for reward sharing when the item is deposited. Transactions that are not included in the blockchain will not be counted for reward sharing, therefore robots must stake in order to receive their recompense for selling information. At reward sharing, the smart contract distributes to all sellers both the reward share and all staked amount. Therefore, when stakes are sufficiently high, robots that sell information classified as outlier lose crypto tokens because the staked amount is larger than the share the smart contract distributes to them. Additionally, information that is not supported by stake (hence not included in the blockchain) must be judged as untrustworthy and robots will not use it for updating their motion path. In other words, stakes are also a form of insurance that the seller sends information that it deems accurate.

**Figure 4:**
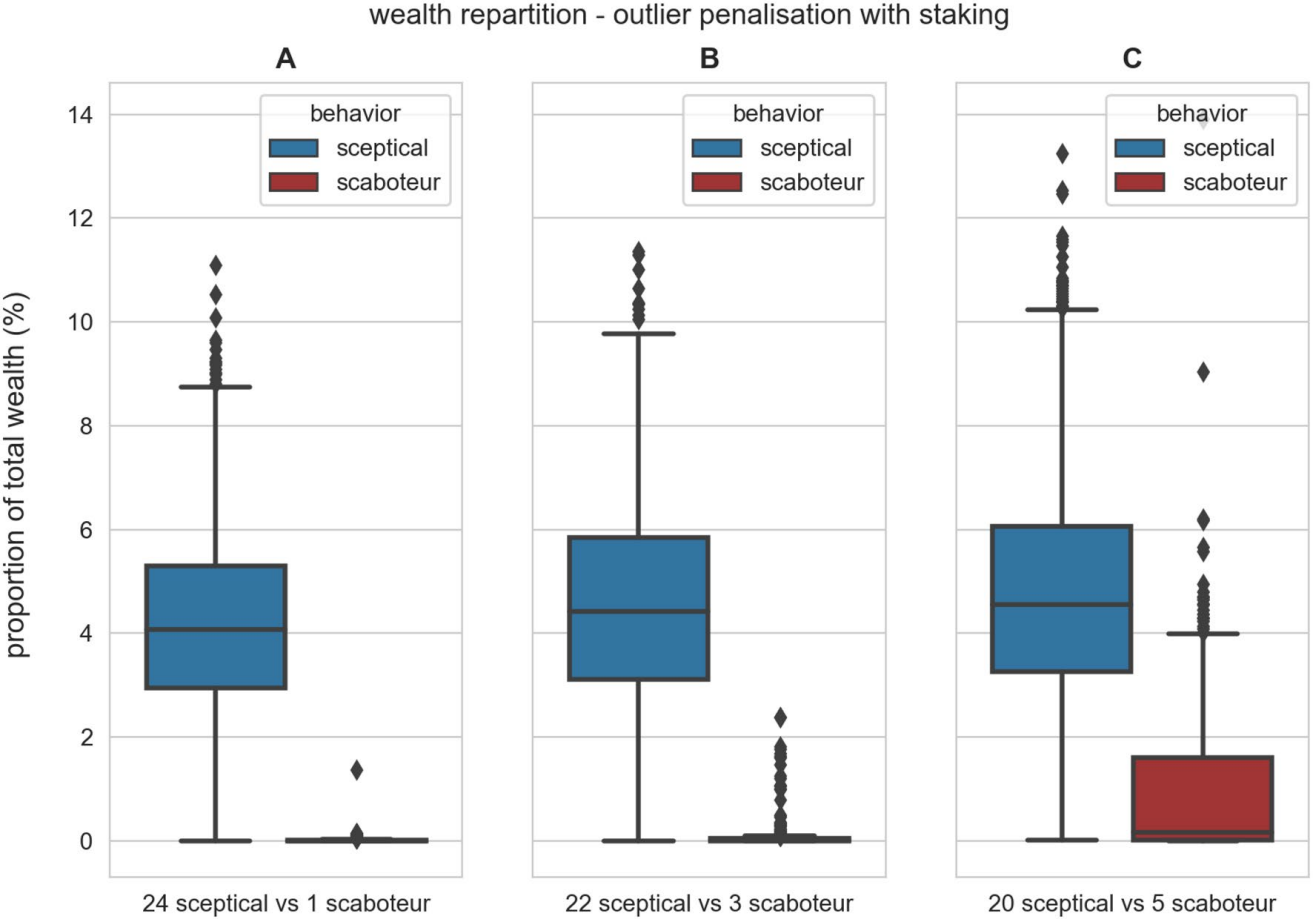
Results of the experiments with the outlier penalisation with staking payment scheme (128 simulations per condition). We varied the swarm composition (indicated under each panel) in swarms composed of 25 robots (see description of the box plots in the caption of Fig. 2). In all tested conditions, the blue distribution (honest robots) is significantly higher than the red one (Byzantine robots); Mann–Whitney *U* test (*p*-value < 0.001 in all cases). Including the staking mechanism reduces considerably the Byzantine robots’ wealth.

**Figure 5:**
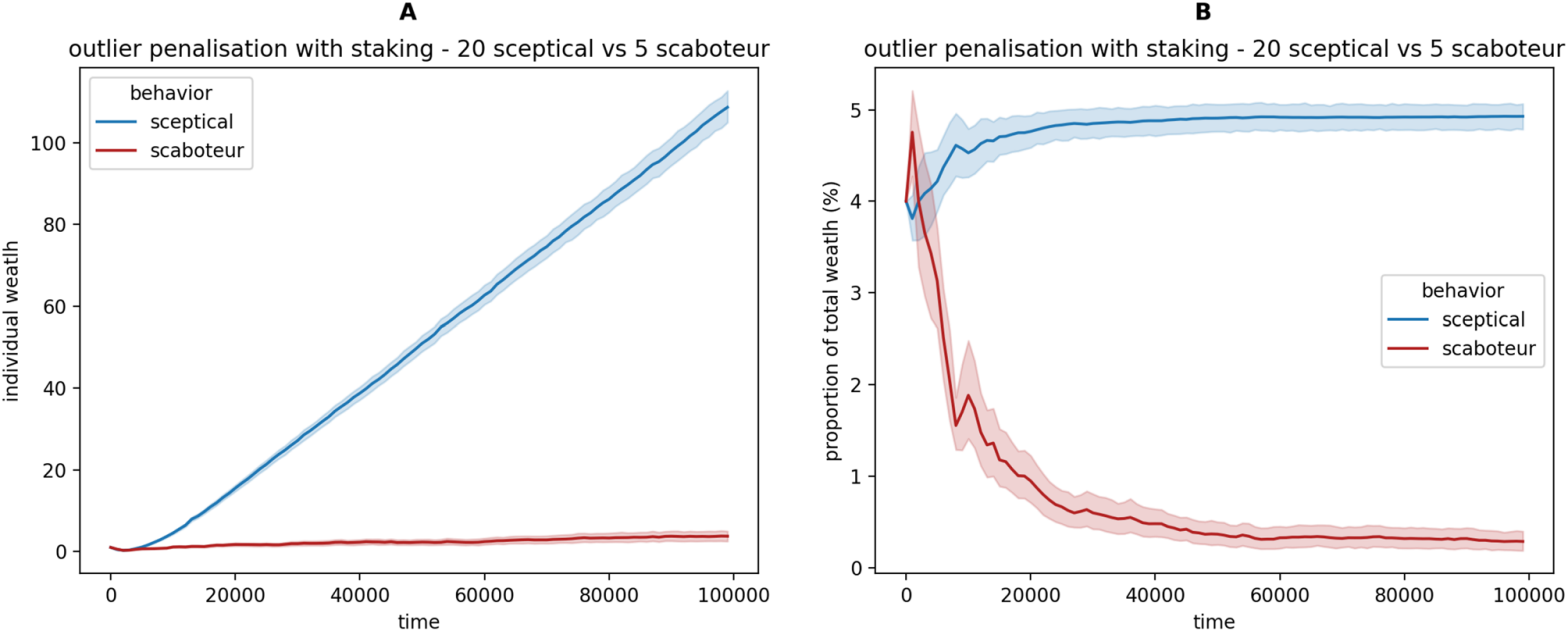
Average robot’s wealth in a swarm of 20 sceptical and 5 scaboteur robots using the outlier penalisation with staking payment scheme in 32 simulations. The transparent shades show the 95% confidence interval. (**A**) The absolute wealth of honest sceptical robots constantly increases at a high pace compared with the scaboteurs that remains with relatively low, almost null, wealth. (**B**) The proportion of total wealth rapidly converges to a relatively stable situation where wealth is mainly distributed among honest robots, approximately equally.

Figure 4 shows that adding the staking mechanism increases the difference in proportion of wealth between honest and Byzantine robots (in comparison with Fig. 3 where staking is not present). Figure 5A shows that, over time, Byzantine robots increase their wealth at a rate considerably lower than honest robots. While the Byzantine robots lose money through staking, they are still able to obtain a reward by transporting items and, in our experiments, foraging gains are higher than staking losses (Fig. 5A). However, the difference in wealth proportion becomes and remains high as the simulation continues (Fig. 5B). Such differences in wealth can be potentially used, in future studies, by the robots to select, depending on the seller’s wealth, which information comes from a trustworthy party and can be used directly, and which information comes from mistrusted sources and therefore they should be sceptical about.

**Figure 6:**
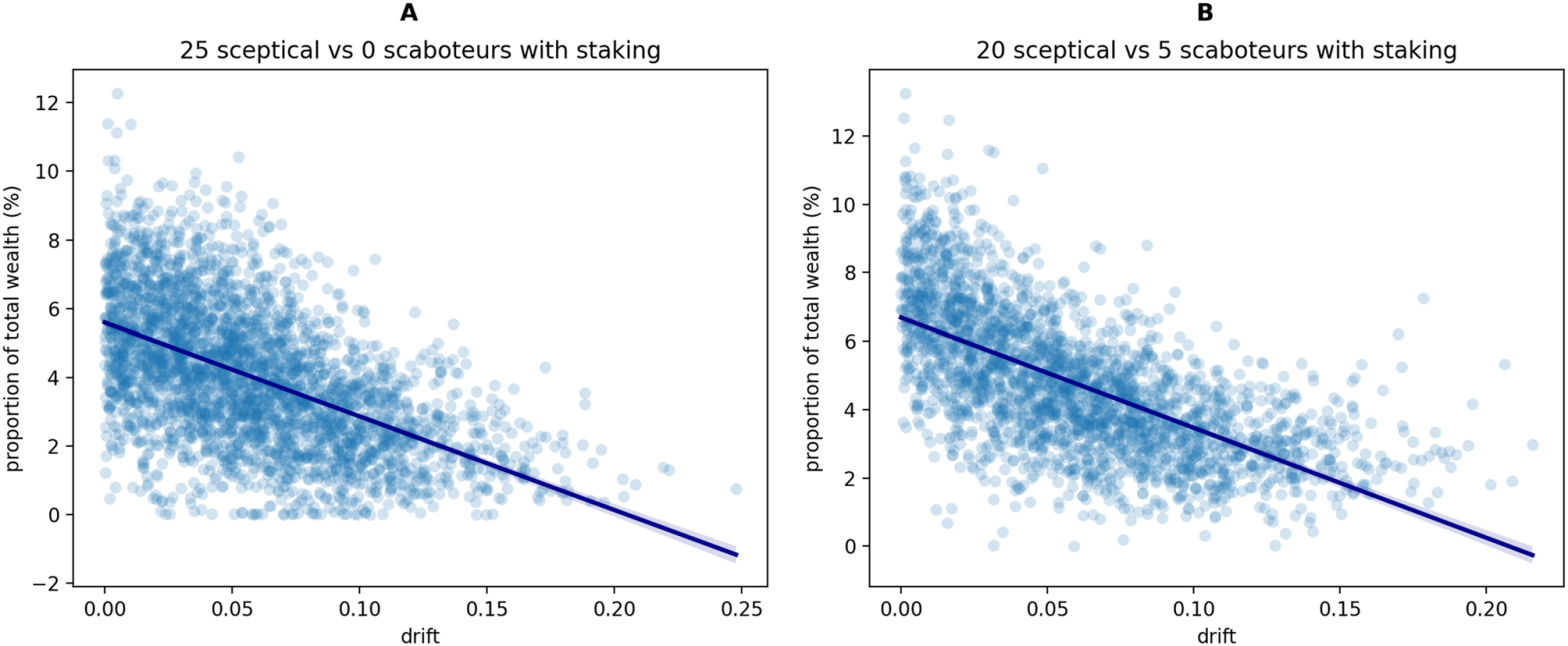
Relationship between odometry error (indicated as the mean angular drift on the *x*-axis) and robot’s wealth (indicated as wealth proportion on the *y*-axis), for swarms of 25 robots after 15 000 timesteps (results of 32 simulations, data reported only for the honest robots subgroup). The two measures are correlated both in case of (**A**) Byzantine-free swarms and (**B**) swarms with 5 scaboteurs, with Pearson correlation coefficients of $-\,0.54$ and $-\,0.64$ respectively (solid lines with the 95% confidence interval shown as transparent shades).

In addition to be used to neutralise Byzantines, robots’ wealth can also be an indication of the robot’s contribution to the task. Wealthier robots have completed more round-trips by accumulating less odometry error and, in turn, sold more accurate information. The results reported in Fig. 6 show that indeed robots subject to lower levels of odometry error are also among the wealthiest after 15 000 timesteps. Through statistical analysis, we found a negative Pearson correlation coefficient between absolute bias in odometry drift and accumulated wealth ($-\,0.54$ for Byzantine-free swarms, and $-\,0.64$ for swarms with 5 scaboteurs). Thus, wealth can also be a candidate metric to measure robot’s navigation efficiency, as well as to attribute credit for task completion.

### Byzantine behaviour

Most of our analysis focuses on protecting the swarm from the Byzantine robots that share navigation vectors rotated by 90 degrees because it is the most efficient strategy to let a naive robot drift away from the robot chain (as the rotated vector is perpendicular to the chain, see also Movie S1). The investigated protection mechanisms have been shown to be resilient to such Byzantine behaviour to different extents. However, it is not evident how the swarm would respond to different Byzantine behaviours. Therefore, we investigated the collective performance against a range of nine Byzantine behaviours. We acknowledge that there can be several other Byzantine behaviours that are not included in our analysis, however the goal of this study is to show the feasibility of an economics-inspired approach for designing Byzantine-tolerant robot swarms, rather than proposing algorithms that can neutralise any conceivable Byzantine behaviour. Developing such algorithms is the long-term goal of the economics-inspired research on swarm robotics that we introduce in this study.

**Figure 7:**
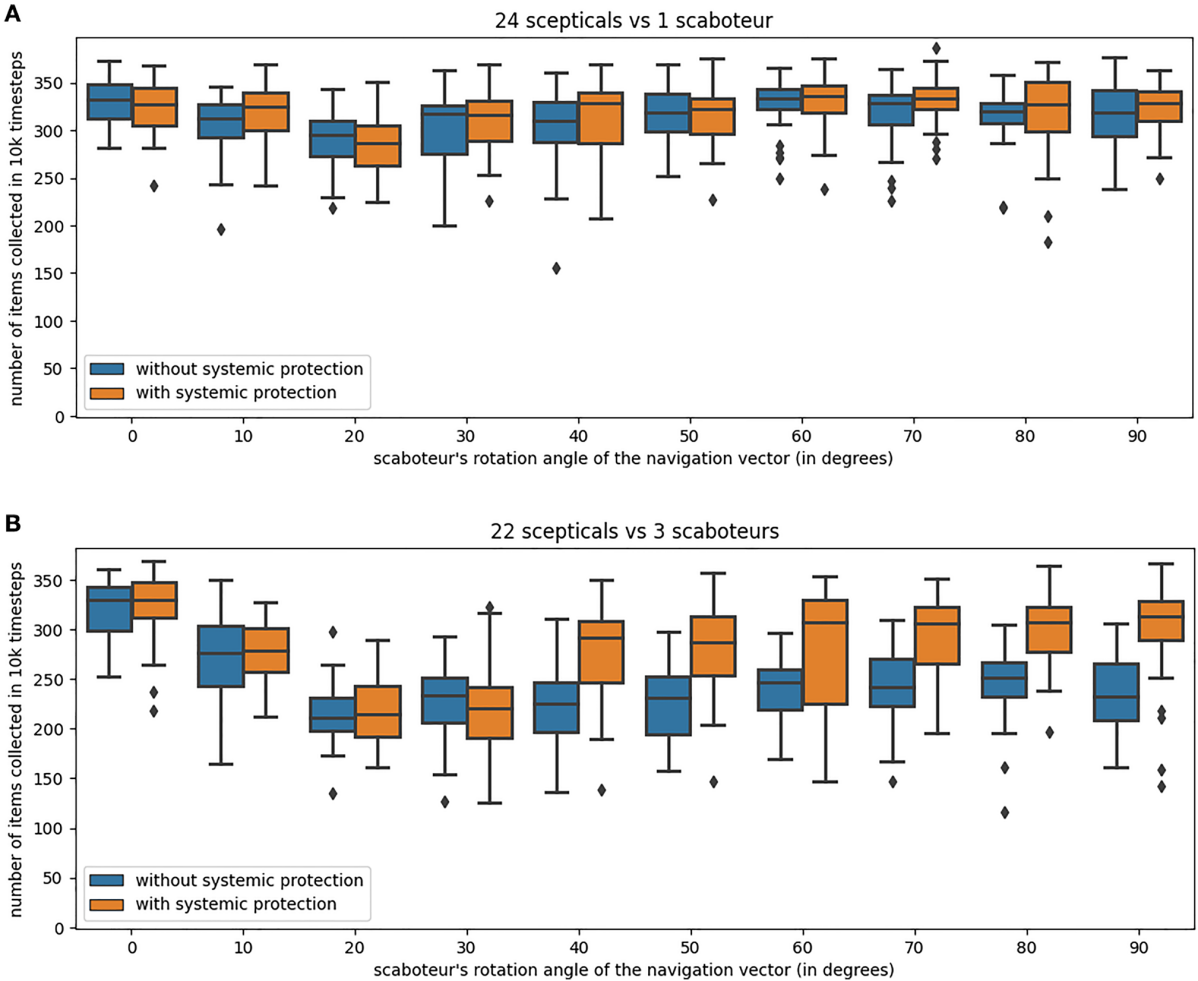
Collective performance comparison between robot swarms that only use individual protection and swarms that use both individual and systemic protection through outlier penalisation with staking. The collective performance (*y*-axis) is measured as the number of items collected by a swarm of 25 robots in the last 10 000 timesteps of an experiment 30 000 timesteps long (results of 32 simulations). We only record the collective performance in the last part of the experiment in order to study the dynamics once the robot chain—connecting nest and food—is formed. The swarm comprises one Byzantine robot (panel **A**) or three Byzantine robots (panel **B**) which consistently send incorrect path information by rotating the navigation vector by the angle indicated on the *x*-axis (note that when the rotation angle is equal to zero there are no Byzantine robots in the swarm).

In Fig. 7, we show the collective performance of a swarm of 25 robots comprising one or three Byzantine robots (scaboteurs) which share navigation vectors rotated by a given angle. The Byzantine behaviour—i.e. the rotation angle—is indicated on the *x*-axis (note that scaboteurs with rotation angle equal to zero have a behaviour equivalent to the non-Byzantine robots). The *y*-axis shows the collective performance as the item throughput over the last 10 000 timesteps. For each tested Byzantine behaviour, we report the collective performance of the system with and without systemic protection based on outlier penalisation with staking. Interestingly, comparing the two systems, outlier penalisation with staking does not bring any apparent advantage when a single Byzantine robot is present (Fig. 7A). Individual scepticism allows the robots to ignore information that is not confirmed by any second robot and, in this set of experiments, it appears to be enough to neutralise the impact of a single Byzantine. Instead, the collective performance is significantly higher with systemic protection based on the information-market economy, than without it, when there are three Byzantine robots (Fig. 7B). Increasing the number of Byzantine robots also causes an increase in the variability of the collected results (i.e., boxes and whiskers are longer in panel B than in panel A). Each simulation is distinct and there are runs with low collective performance because robots with particularly high odometry noise do not receive frequently enough new navigation information; this happens more frequently when the number of robots maintaining the chain between nest and food (i.e., non-Byzantine robots) are lower (24 robots in panel A and 22 in panel B). Additionally, individual scepticism rules are designed to let a robot accept information that is confirmed twice; therefore, in setups with more Byzantine robots, the probability of sporadically accepting false information increases (leading to a temporary destruction of the robot chain connecting food and nest). In future work we intend to study how to improve the scepticism rules by filtering path information based on the wealth (or reputation) of the robot providing information. Nevertheless, the results of Fig. 7 already show that systemic protection inspired by economic mechanisms implemented on blockchain smart contracts can protect the swarm from Byzantine behaviour.

When comparing the different Byzantine behaviours, Fig. 7 shows that their impact on the system with systemic protection is approximately the same when the rotation angle is greater than the threshold $\Theta =30^\circ$ used to classify outliers by the smart contract. Instead, when the rotation angle is smaller than $\Theta$, the Byzantine robots are able to deteriorate the collective performance because sporadically a few robots drift away from the robot chain connecting food and nest. Even though in this study the smart contract classifies (and rewards) navigation vectors as similar or different based on a fixed threshold $\Theta$, thus making it easy for malevolent Byzantine robots to exploit this smart contract weakness, future work will investigate how to strengthen the outlier classification algorithm, for example, by changing $\Theta$ adaptively as a function of the reputation of the robot providing information. In addition, while the transparency of blockchain smart contracts (which are algorithms with code and data publicly available^19^) may enable exploitation by Byzantine agents, in a real-world deployment, such an open-source system facilitates detecting flaws in the economic mechanism and subsequent correction (Linus’s law^56^). Our approach to open swarm robotics seeks a balance between an accessible knowledge of the global economic rules and the use of cryptographic methods that can ensure privacy and protect individual robots from targeted attacks.

## Discussion

This article introduces the concept of an information market where robots can buy and sell information instead of simply exchanging it for free. Through multiagent simulations, we provide a proof of concept for an information market regulating robots’ social navigation during the execution of central place foraging. We show that through economic transactions (and potentially smart contracts) we can design robot-to-robot payment schemes and reward mechanisms that redistribute wealth from robots sharing directions deemed as outliers to robots sharing directions that fall in line with information shared by the majority of the swarm. Our economic rules succeed in penalising dissemination of incorrect information and, in turn, encourage the exchange of truthful information. Being this a proof of concept aimed at proposing a new economics-inspired approach for the design of robot swarms, the case study is relatively simple and has some limitations which can be addressed in future work. For example, the environment consists of an obstacle-free plane with two sites only (food and nest), the robots are simulated as point particles without collisions, and the exchanged path information is a simple vector. While the simplicity of our setup allowed us to show the strength and potential of the economics-inspired approach, future studies developed with physical robots navigating through complex environments and exchanging paths based on elaborated trajectories (e.g., using waypoints) will be able to confirm the possibility of employing our new approach to deploy resilient robot swarms in the real world.

There are several related studies that employ control theory to implement resilient policies for different multi-robot systems^8,11,57–61^. Such solutions are effective methods to protect the system from a variety of Byzantine attacks and have been validated on a variety of multi-robot systems. However, the majority of this research has not addressed *open* multi-agent systems or *open* robot swarms; by open robot swarms we mean distributed open-participation systems composed of robots that are potentially deployed by different owners^4,13^. Hence, the swarm can be heterogeneous, comprising robots with different abilities and specifications, and built and programmed by different producers. It is acknowledged that the “openness of such systems negates many traditional security solutions”^62^ and addressing security issues in open systems is in its infancy^61^. One of the main challenges in open systems is the resistance to Sybil attacks (i.e., to attacks performed through multiple fake identities) because, without this core feature, agents performing the Sybil attack can gain disproportionate power in the network and nullify the protection from other security strategies. Therefore, achieving security and collaboration among (potentially self-interested) robots in open robot swarms is a critical challenge. Blockchain technology has been identified as one of the most promising solutions to protect open systems from Sybil attacks^63^. Our research suggests a blockchain-based solution for controlling the collective behaviour of open robot swarms based on the design of a robot economy where robots have incentives to cooperate with one another and are economically penalised for hampering task execution. In addition, robot swarms regulated by economic mechanisms can prevent unbounded duplicated identities while accepting contributions by new members, as long as they adhere to the economic rules.

Having a tamper-proof shared knowledge in a decentralised network of nodes that cannot trust each other has only been possible with the relatively recent invention of blockchain technology^64^. Indeed, according to the current state of the art, blockchain is the only technology that allows recording economic transactions and execute tamper-proof algorithms in a fully decentralised and secure manner. Our simulations are intended to be transferred to robot swarms in which every robot is a blockchain node—similar to the systems proposed by Strobel et al.^4^ and Pacheco et al.^17^. Although the technical implementation of a blockchain system is outside the scope of this work, our simulations are designed to model the relevant aspect of a blockchain-based system. In particular, the database that stores payments and rewards is structured to emulate the functionalities of a blockchain supporting smart contracts, and the robot behaviours are designed around the transaction-based nature of peer-to-peer interactions in blockchain networks. To prove the realism of our simulations and the possibility, in the future, to implement an information market on real robot swarms based on our approach, we developed a smart contract and the entire communication protocol to allow path information exchange and reward sharing. The smart contract—written in the programming language of Ethereum smart contracts, Solidity—can be found in the *Supplementary Information*. In our implementation (presented in detail in the *Methods* section), information exchanges are based on a combination of off-chain contracts, named as *light contracts*, (i.e. the robots directly exchange cryptographically-secured information without relying on the blockchain) and on-chain transactions (using a blockchain smart contract).

Our study differs from previous work^3,4,17,15^ that employed blockchain technology to allow robots to collectively add information to a shared knowledge database, filtering out incorrect information. In this paper, robots are not simply moving sensors that use the blockchain to securely store and merge their individual readings; rather, they are robots that are meant to be economic agents acting in a physical environment and that use the blockchain for one-to-one economic transactions. In fact, in our foraging scenario, information stored in the blockchain and exchanged between robots is only relevant to the robots involved in the information transaction (i.e. the buyer and the seller), and not to any other robots. The path information is expressed in the buyer’s relative coordinate system, and is therefore useless to other robots. Nevertheless, storing the information on the blockchain enables the creation of an information market that fosters collaboration among self-interested parties. Similarly to most swarm robotics systems, in our study, exchange of information is critical to efficient task execution and our information market provides incentives for such information exchange to happen. In our information market, the blockchain has the double role of keeping track of every exchange of crypto tokens among robots (i.e. distributed payment ledger) and to apply system-wide rules (i.e. economic payment schemes) in the form of smart contracts. For this reason, despite being useless to other robots, pairs of robots exchange information via the blockchain so that the smart contract can apply an outlier detection algorithm and distribute rewards accordingly. Regulating information exchange through economic rules proves to increase the benefits of honest cooperation, penalises mistakes or deliberate spreading of misinformation, and, in turn, increases the foraging efficiency of the whole swarm.

Our study showcases the viability of economics-inspired swarm robotics, a promising, yet unexplored, paradigm with high potential. In human societies, collaboration towards common goals is often dictated by economic factors that set incentives for cooperative behaviour. For such systems there is a large body of literature that can be the source of insightful mechanisms to prevent self-interested individuals to hamper collective success. For centuries, the only way to guarantee that all parties obeyed the systemic (economic) rules has been to rely on central authorities—i.e. banks and governments—enforcing the rules and punishing rule breakers. Therefore, such an architecture, based on central authority, has never been considered as a suitable inspiration source for the design of robot swarms, characterised by their distributed nature and therefore by the absence of a central component. Only a few years ago^18,64^, the blockchain revolution introduced the possibility to enforce system-wide rules in a fully decentralised and trustless network—i.e. a network in which participants do not need to trust each other nor a central authority. By regulating information exchange through blockchain technology, swarms of self-interested robots can remain fully decentralised and at the same time their constituent robots can trust the information received by others without the need of a third party acting as a guarantor or rule enforcer. Hence, we suggest to take a similar approach to what gave rise to the research field of swarm robotics^65^, which is rooted in the biological-inspiration from eusocial insects, or collective animal behaviour in general. Taking inspiration from economics to design decentralised open swarms is a research direction that we believe to be at the beginning of a long fruitful journey.

## Methods

Our study is based on multiagent simulations which keep the application scenario and several implementation aspects at an abstract level. The simplicity of our simulations allows us to focus on the individual behaviours and the economic rules that can enable secure cooperation in swarms of self-interested robots. Our simulator is open-source (https://github.com/ludericv/information-market) and structured in a way to allow future users to easily extend both the robot behaviour and the economic rules. Our design implementations have been motivated by making the simulator accessible to scholars from domains with limited programming experience who would like to test how to secure robot swarms with new economic mechanisms.

Here, we first present the basic structure of the simulator, comprising the environment and the basic motion behaviour of the robots. Then, we show the simulation modules that a user can customise to define what information to buy and to sell as well as how to use social information; in our case, we present all robot behaviours investigated in this study. We continue by presenting how we implemented the simulated blockchain and the smart contracts. Finally, we provide a detailed description of how the smart contract can be implemented in Solidity—an object-oriented programming language for implementing smart contracts on Ethereum.

### Basic structure of the simulator

**Simulator architecture** As shown in the UML diagram of Fig. S2, the simulator separates the code defining the simulated environment, the simulated robot’s hardware (sensors, actuators, communication) which is fixed for all robots, the robot’s behaviour which can be customised for every robot, and the simulated blockchain and smart contract functions.

**Environment** In our simulations, the robots move in a finite 2D rectangular arena of size $W \times H = 1200$ units $\times \ 600$ units. This arena is empty except for two specific sites that are placed symmetrically to the left and right of the environment: the food site and the nest site, respectively located at $P_F = (200, 300)$ and $P_N = (1000, 300)$. These sites are circular areas with radius $r_F = r_N = 50$ units.

**Robot’s motion and sensing** The robots have to transport items from food to nest. Robots are modelled as point particles that move at speed $v=2.5$ units per time step and can rotate instantaneously. Robots can only detect the position of a site when they are inside it (i.e. from a distance of $r_F=r_N=50$ units from the site’s centre); however, they do not have access to any global positioning device (e.g., a GPS) to accurately know the absolute position of the site nor their own position while moving throughout the environment. Therefore they resort to odometry, by updating each site’s relative position after every movement.

**Odometry noise** When a robot detects a site, it stores its exact position which updates at every movement through odometry which however is subject to measurement noise. The accumulation of odometry noise leads robots to drift away from the trajectory they think to be following. In our simulations, each robot has two noise parameters: bias $\mu$ and standard deviation $\sigma$. At each time step, a robot sets its desired movement as a vector in its own reference frame. However, its actual movement is rotated by an angle sampled from a normal distribution with mean $\mu$ and standard deviation $\sigma$. We assume the robot uses odometry readings that indicated the desired movement was carried out perfectly, therefore, there is a discrepancy between the robot’s recorded movement and its actual motion. As a result, a robot that thinks to move perfectly straight will actually be moving on a curved trajectory, as it is on average turning $\mu$ degrees each step.

While the standard deviation $\sigma$ is equal for every robot (in our simulations $\sigma =0.05^\circ$), the bias $\mu$ is distinct for each robot. At the beginning of each simulation, each robot’s $\mu$ is drawn from a a bimodal distribution $\mathcal{K}(m_{\mu }, s_{\mu })$﻿ of the following form:

1 $$\begin{aligned} \mathcal {K}\left( m_{\mu }, s_{\mu }\right) = \frac{1}{2}\left( \mathcal {N}\left( m_{\mu }, s_{\mu }\right) + \mathcal {N}\left( -m_{\mu }, s_{\mu }\right) \right) \end{aligned}$$

where $\mathcal {N}\left( m_\mu , s_\mu \right)$ denotes a normal distribution of mean $m_\mu$ and standard deviation $s_\mu$; in our simulations $m_\mu =s_\mu =0.05^\circ$. This means certain robots have a higher odometry noise than others and thus accumulate larger errors.

Our case study relies on information exchange to perform cooperative navigation and collectively filter out the individual errors. Therefore, sensing and actuation noise is a crucial aspect of our study, and it is therefore important to consider in the simulation robots that have different error levels as this can be a crucial component of the resulting collective behaviour^66^. For this reason, we simulate robots with distinct random levels of odometry noise. Note that, although the specific noise level is normally unknown to the robot and its users, our economics-based framework provides a way to easily and transparently sort the robots according to the magnitude of their measurement and movement errors (Fig. 6).

**Navigation table** Each robot stores path information in a navigation table with two entries (see Table 1). Each entry corresponds to a site (i.e. food or nest) and consists of four attributes: the site type (food or nest), a vector indicating the estimate of the relative position of the site in the robot’s local coordinate system, the age of the information, and a Boolean flag indicating if that information is still valid. After each movement, robots update their navigation tables by changing the relative position estimates according to their odometry estimate, and by increasing the corresponding age by one. The “valid information” flag is set to False if the relative position’s Euclidean norm becomes smaller than the robot’s sensing range $r=8$ and the site is not sensed. When a robot’s target location is unknown (i.e. no valid information is available), robots can only explore the environment randomly. Robots can acquire valid information either from other robots or by sensing a site. Once a robot is within a site (i.e. at a distance smaller than $r_F = r_N = 50$ units from its centre), it can update the navigation table by storing the exact relative position of the site’s centre and set the information age to 0.

**Table 1:** Example of a navigation table used by each robot to store its best estimate of the relative position of the two sites.

Site type	Relative position	Age	Valid information
Food	$(200, -\,5)$	78	True
Nest	$(-\,1000, 35)$	400	True

**Random exploration** When a robot has no valid information to reach a site, it explores the environment through a random walk. In a random walk, two main characteristics of the movement are subject to randomness: the turning angle $\phi$, and the amount of time or distance travelled between consecutive turns (also called step-length $\delta$)^67,68^. For the experiments conducted in this article, the search strategy is a hybrid between a correlated random walk and a Lévy walk, which previous research has identified as the most efficient strategy to locate sites in an unknown environment^67^.

A correlated random walk means that there exists a correlation between a robot’s consecutive turns, such that a robot is more likely to continue moving in the same direction, in other words, it is biased toward low amplitude turning angles. A probability density function having such characteristics is a wrapped Cauchy distribution, which reads as:

2 $$\begin{aligned} f_{\rho }(\phi ) = \frac{1}{2\pi }\frac{1-\rho ^2}{1+\rho ^2-2\rho \cos \phi }\,, \end{aligned}$$

where the parameter $0<\rho <1$ defines the skewness of the probability distribution. In a pure correlated random walk, the step-length $\delta$—i.e. the time between consecutive turns—follows a normal distribution.

A Lévy walk is characterised by its heavy-tailed step-length distribution, following a power law,

3 $$\begin{aligned} P_{\alpha }(\delta ) \sim \delta ^{-(\alpha +1)}\,,\, \text { with } 0<\alpha \le 2, \end{aligned}$$

which in practice leads to series of quick turns (allowing local exploration of an area) followed by long straight-line displacements. A pure Lévy walk has a uniform turning angle distribution.

The random walk implemented for the simulations has a distribution of turning angles according to the correlated random walk (with parameter $\rho =0.9$), and a Lévy step-length distribution (with parameter $\alpha =1.4$).

**Load/unload time** In order to simulate the time that would be necessary to physically load or unload an item, the robots spend a variable amount of time within each site. Including variable load/unload time at every site makes our simplified simulation more realistic and also removes the formation of robot platoons, where robots have fixed relative positions with one another. In fact, robots that move in a dynamic chain between the two sites, have a constant and equal load/unload time, and move at the same speed, will maintain their relative position with respect to the other robots throughout several round-trips. Instead, in our simulations, the time spent by the robots in each site is variable as the robots have to reach a random point, chosen uniformly within the site, every time they want to collect or deposit an item in the food or the nest sites, respectively.

**Communication** Robots can communicate with other robots in a communication range $r_C=50$ units, depicted as gray circles in Fig. 1. Robots exchange messages in order to buy and sell path information. The decision of which information to buy and to sell and of how to use such information is implemented in the robot behaviour described in the next section (see also the *Behaviour* class in Fig. S2).

**Swarm size** We ran experiments with swarms comprising 25 simulated robots. Robots are able to collectively filter individual odometry noise by exchanging path information based on an existing social odometry algorithm^29,43–45,53^. Depending on the level of odometry noise, the distance between the sites, and the communication range, the swarm size influences the collective foraging performance. The larger the swarm is, the more frequent the encounters providing social information are and, in turn, the robots are better at filtering out odometry noise, improving their navigation. However, swarms comprising a very large number of robots moving in a finite space can cause physical interference undermining the collective accuracy^30^. Therefore, the collective foraging performance of this social navigation system follows a typical scalability curve^69^, which increases with the swarm size when the number of robots is moderate and decreases with the swarm size once the space is saturated and movement is congested. While our simulations do not consider physical interference and therefore the performance would increase monotonically with the swarm size^70^, in our experiments we kept a reasonable number of robots (25 robots), so that they do not overcrowd the environment.

### Robot behaviours

The robot behaviour (*Behaviour* class in Fig. S2) defines how information is used and shared through three methods:

1. step: this method defines the desired robot’s movement, based on the available information;
2. buy_info: this method defines what information to buy and how to combine it with the information in the navigation table (Table 1);
3. sell_info: this method defines what information to sell to other robots;

We present four robot behaviours: naive, sceptical, saboteur, and scaboteur.

**Naive behaviour** The naive robots constantly move at maximum speed $v=2.5$ units per time step between food and nest following the information stored in their navigation table. If the navigation table’s information is not valid they perform a random walk.

Every robot broadcasts at every timestep the age of the (valid) information in its navigation table. The naive robots decide to buy information for a given site when the age of the information they can buy is lower than the age they have in their navigation table for that site. Then, the buyer combines the bought information with the one it already has in its navigation table using a weighted average as follows:

4 $$\begin{aligned} \mathbf {\textbf{x}}&= \frac{a_{buyer}}{a_{buyer}+a_{seller}} \cdot \mathbf {\textbf{x}}_{seller} + \frac{a_{seller}}{a_{buyer}+a_{seller}} \cdot \mathbf {\textbf{x}}_{buyer}\,; \end{aligned}$$

5 $$\begin{aligned} a&= \frac{a_{buyer} + a_{seller}}{2}\,, \end{aligned}$$

with *a* representing the new navigation table entry’s age attribute and $\mathbf {\textbf{x}}$ its 2D relative position vector. Since robots only buy more recent information, we have $a_{buyer} > a_{seller}$, therefore more importance (i.e. a higher weight in Eq. (4)) is attributed to the new information without completely discarding the previous one with an outright replacement. The decision to only buy information with lower age is motivated by the fact that the age indicates the number of odometry updates that have been applied to that piece of information, each time including an error, thus robots only acquire information with lower expected error than the information they already have. The decision to combine the two pieces of information with a weighted average as from Eq. (4) is motivated by the analysis described in Text S1 in the *Supplementary Information*, where we tested alternative methods for updating the navigation table’s information (e.g. replacement, averaging, noise estimates) and Eqs. (4)–(5) showed the best performance.

The naive robots always sell to other robots the values they have in their navigation table.

**Saboteur behaviour** The saboteur behaviour is based on the naive behaviour and uses the same methods for movement (step) and for using other’s information (buy_info). However, saboteur robots, when selling information (sell_info), they rotate the vector they have in their navigation table by 90 degrees.

**Sceptical behaviour** The sceptical behaviour is also based on the naive behaviour and uses the same logic to move in the environment (same step method) and to sell information (same sell_info method). The sceptical behaviour differs from the naive behaviour by how the robots use social information (buy_info method). Sceptical robots decide to buy information using the same condition as indicated in the naive behaviour (i.e. any information with lower age); however, before updating the navigation table with Eqs. (4)–(5), the robot performs an additional verification step. This step aims to make the robot less susceptible to information from saboteur robots. When new information is bought, it is added to a pending information table and compared with the entry in its navigation table for the same site, as well as to the other entries on the pending information table. The comparison of each pair gives a *difference score* computed as

6 $$\begin{aligned} \text {diff}(i, j) = \frac{||\mathbf {\textbf{x}}_i - \mathbf {\textbf{x}}_j||}{||\mathbf {\textbf{x}}_i||} \end{aligned}$$

where $\mathbf {\textbf{x}}_i$ and $\mathbf {\textbf{x}}_j$ are the relative position vector attributes for the preexisting and just-bought information, respectively, and ||...|| denotes the Euclidean norm of a 2D vector. When the difference score is lower than a threshold $\lambda =0.25$, the just-bought information is used to update the navigation table entry (using the weighted average computation of Eqs. (4)–(5)).

**Scaboteur behaviour** The scaboteur behaviour is a combination of the sceptical and the saboteur behaviours. The Scaboteur robots use the same methods for movement (step) and for using other’s information (buy_info) as the sceptical robots. And, in the same way as saboteur robots, scaboteurs sell to other robots vectors rotated by a given angle. In most experiments, the angle of rotation is fixed to 90 degrees, so that the resulting rotated vector is perpendicular to the direction of motion of the robots in the chain connecting food and nest, and, in this way, it can efficiently divert robots away from the chain.

### Simulated blockchain and smart contracts

In our experiments, the blockchain is simulated as a shared database that can be updated by the robots by creating transactions, which are blockchains’ data entries. These transactions are used to store information in the databases as well as to keep track of the wealth of every robot. All robots start the experiment with a wealth of 3. There are two types of transactions: one is created when robots exchange path information and the other when the item is deposited and the reward is shared among robots. We present the two transactions for the two payment schemes considered in this study.

**Outlier penalisation scheme** In the outlier penalisation payment scheme, every time a robot sells path information on how to reach a site to another robot, it creates a transaction that stores information on the simulated blockchain. Each transaction contains the buyer’s and seller’s unique IDs, the site type (nest or food), and the 2D vector pointing to the site in the buyer’s coordinate system.

When the robot *i* deposits an item in the nest, a reward $R=1$ is issued. The depositing robot receives *R*/2, while all *contributors*—i.e. the robots that provided information to robot *i*—receive a share of the remaining *R*/2. Contributors to *i* are computed by the simulated smart contract as all the robots that sold information to *i* (stored in previous transactions of the shared database). Note that contributors are computed only for the last round trip of *i*, i.e., only for the time after the previous reward received by *i*. For each transaction, a contributor receives $w_x\frac{R}{2}$, where $w_x \in [0,1]$ is a weight computed as,

7 $$\begin{aligned} w_x&= \sum _{y = 1}^{|T|} \text {similar}\left( t_x, t_y\right) \,/\, \sum _{k = 1}^{|T|} \sum _{y = 1}^{|T|} \text {similar}\left( t_k, t_y\right) \end{aligned}$$

8 $$\begin{aligned} \text {similar}\left( t_x, t_y\right)&={\left\{ \begin{array}{ll} 1, &{} \text {if }|o_{t_x} -o_{t_y}| < \Theta \text { AND }\text {site}_{t_x} = \text {site}_{t_y}\\ 0, &{} \text {otherwise}\,, \end{array}\right. } \end{aligned}$$

where *T* is the set of the recorded transactions in the simulated blockchain for the last round trip. The weight $w_x$ of transaction $t_x$ counts the number of similar transactions according to the function similar$\left( t_x, t_y\right)$ which determines whether transactions $t_x$ and $t_y$ are considered similar, i.e. regard the same site, and the vectors’ orientations *o* differ by less than $\Theta =30^\circ$. Since the transactions with information from saboteurs have an orientation *o* largely different from honest information, their weight $w_x$ is on average lower, yielding lower rewards. The denominator on the right-hand side of Eq. (7) normalises the values of $w_x$ so that the weights of all transactions in *T* sum to 1.

**Outlier penalisation with staking scheme** This payment scheme extends the outlier penalisation by including the staking mechanism when path information is sold from one robot to another. The selling robot, when it creates a transaction (which will be later used to distribute rewards), must stake an amount $\epsilon$ of crypto tokens (in our experiments, we used $\epsilon =0.04$). The staked tokens are locked and not available to be spent by neither the buyer nor seller of the information. The staked tokens will be then released and distributed among all contributors in the same way as done with the shared reward in the outlier penalisation payment scheme. Therefore, for each transaction, a contributor receives $w_x\left( \frac{R}{2}+|T|\epsilon \right)$ (where |*T*| are the transactions recorded in the last round trip as from Eq. (7)).

### How to transfer our solution to a blockchain-based smart contract

**Figure 8:**
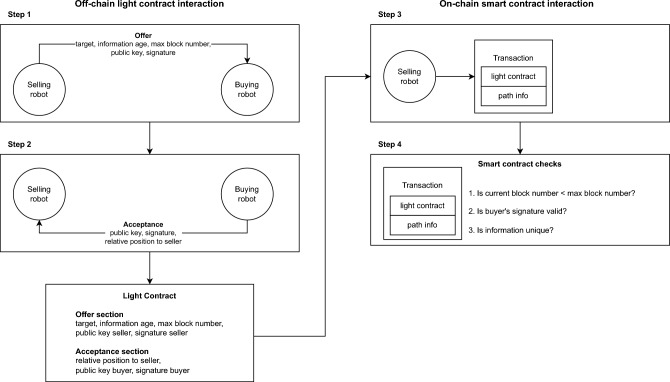
The process of information exchange between the selling and the buying robots comprises four steps. **Step 1**: A selling robot makes an offer to a buying robot. This offer includes the target (food or nest), information age, and a maximum block number which specifies when the light contract must be included in the blockchain at the latest. **Step 2**: If a buying robot agrees on the offer, it returns its signature of the offer and its corresponding public key. In addition, it returns its relative position to the selling robot. Both parties now possess a double-signed light contract. The light contract is an off-chain agreement between two robots that is fast to perform since it does not require a transaction on the blockchain network. **Step 3**: The selling robot creates a blockchain transaction that includes both the light contract and the now revealed path information to the target site in the buyer’s reference frame. **Step 4**: The smart contract performs three security checks before it adds the transaction to the blockchain. These checks ensure that the terms of the light contract were not violated. If one of the checks fails, the transaction is discarded.

In order to show that both the communication protocol and the payment schemes implemented in our simulations can be effectively translated into a real blockchain-based robot swarm, we design and implement a light contract (a peer-to-peer agreement that occurs off-chain, but is secured by cryptography) and a smart contract that, in combination, enable robots to exchange information and receive crypto token rewards (see Fig. 8). Here, we explain in detail the design of the two contracts and how the robots can securely exchange path information, as well as how the blockchain smart contract can compute reward shares and distribute wealth to the robots. In Movie S2 in the *Supplementary Information*, we showcase an example of such an information exchange.

**Figure 9:**
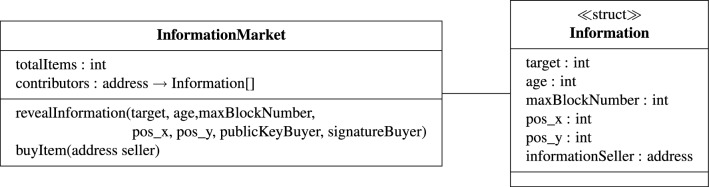
UML diagram of the developed smart contract. The smart contract enables the robots to buy and sell information and the market to buy items. The variable contributors is a mapping from robot addresses to a list of the Information structure. This mapping ensures that the smart contract keeps track of who sold which information to whom; the mapping is used for rewarding all contributors when the item is deposited in the nest and the market buys it.

**Making an offer to sell path information** At any point in time, each robot offers to sell its path information to other robots in physical proximity (Fig. 8—Step 1). These selling offers take place *off-chain* which ensures that information is exchanged fast and exclusively locally (thus, it also removes the need for storing information on the blockchain that is not strictly necessary for the execution of the smart contract).

For making such an offer, the selling robot specifies the target site (either food or nest) and the corresponding age of the information. The offer does not, however, reveal the path information yet, otherwise, the selling robot would run the risk that the buying robot exploits the information without paying or sharing the foraging reward. In addition, the offer contains the maximum block number after which the offer is no longer valid. This number prevents that the information is retained for too long by the selling robot (see more detailed description below). The selling robot signs the offer with its private key (which corresponds to the public key that it uses on the Ethereum blockchain) and sends the signature together with its public key to all robots in its proximity (which are potential buyers). Signing the offer ensures that both the seller identity and the offer’s content cannot be corrupted by a third party (e.g. an impostor).

**Accepting an offer** In order to accept an offer, the buying robot first verifies that the digital signature of the selling robot is valid. If this is the case, the buying robot digitally signs the offer and returns its signature and the corresponding public key to the selling robot (Fig. 8—Step 2). In addition, it returns the seller’s position in the buyer’s coordinate frame including the corresponding signature. Such relative position information is necessary for the seller to compute the path information in the buyer’s coordinate frame.

Both parties are now in possession of a double-signed *light contract* which corresponds to a mutual agreement to exchange path information. The light contract consists of the following information: target site, information age, maximum block number, hashed path information, seller’s position in the buyer’s coordinate frame, public key and signature of the selling robot, and public key and signatures (for both the offer and the seller’s position) of the buying robot. We call this contract a *light contract* because the parties agreed upon it outside of the blockchain (i.e. off-chain), and will only become permanent after being processed by the on-chain smart contract.

**Selling information** Thanks to the buyer’s signature, the selling robot can be certain that it will receive a part of the reward when the buying robot will deposit the item in the marketplace. Therefore, the selling robot is motivated to store the light contract in the blockchain as soon as possible and reveal the actual path information at the same time; this is done by sending an on-chain transaction to the smart contract, which can be immediately seen by the buying robot (Fig. 8—Step 3). This transaction specifies the following arguments for the function revealInformation (Fig. 9): the light contract and the revealed path information. The selling robot reveals the path information in the coordinate system of the buying robot. To do so, the selling robot transforms its path information using the coordinates provided by the buying robot in the light contract (“seller position in the buyer’s coordinate frame”) and its sensor reading of the buying robot’s position (“buyer position in the seller’s coordinate frame”).

To verify the validity of the revealInformation transaction, the smart contract performs three checks (Fig. 8—Step 4). The first check consists of verifying if the agreed-upon maximum block number is less than or equal to the current block number. This check ensures that the selling robot broadcasts the transaction fast enough and, thus, the path information is timely delivered to the buyer. Without a check on the maximum block number, a dishonest seller can delay adding the transaction in the blockchain making the information obsolete and useless to the buyer, while still receiving a share of the reward. The second check consists of verifying if the buyer’s signature of the light contract is valid. This check ensures that the buyer agreed on receiving the path information from the seller in exchange for a share of its future reward. The third check consists of verifying if the combination of target, information age, maximum block number, and buyer/seller pair is unique. This check prevents the seller from reusing the buyer’s signature for another smart contract transaction.

If all checks—maximum block number, buyer’s signature, and unique information—are passed, an Information structure is created and added to the buying robot’s list of Information structures. These structures serve, on the one hand, to provide the path information for the buying robots (i.e. send social odometry information; see paragraph “Accessing information”), and, on the other hand, to reward the sellers for providing information (see paragraph “Reward mechanism”).

**Accessing information** Every time a new light contract is signed, the buying robot waits until it *receives* the path information via the revealInformation transaction (either in a new block or in the memory pool of open transactions). It is important to note that the buyer does not have to wait until the transaction is included in a block, since the hashed path information and digital signatures of the light contract prevent a double-spend attack (i.e. the selling robot cannot create another conflicting transaction). Therefore, introducing a blockchain only causes minimal delay to the presented approach.

**Reward mechanism** Robots are tasked with collecting and transporting items from the food site to the nest site. Upon deposition of the item in the nest, the robot receives a reward. The reward is paid by the *market*—a node on the blockchain network—which recompenses the robot’s work with a reward in crypto tokens. To buy the item, the market creates a buyItem transaction, which transfers crypto tokens to the robot’s account (Fig. 9). In addition—depending on the used strategy—every robot that sold information to the successful forager (i.e. the contributors) gets a share of the reward: through the revealInformation function, the smart contract gets the list of the robots that contributed to the collection of the item by sharing path information. The reward shares are distributed to all contributors by sending the amount of crypto tokens computed by the smart contract to the robots’ public addresses.

### Supplementary Information

Supplementary Information.

Supplementary Video 1.

Supplementary Video 2.

## Data Availability

All data analysed during this study can be generated using the open-source code available in the Zenodo repository https://doi.org/10.5281/zenodo.8187233, which includes a set of scripts to generate all the data used for the figures of this article.

## Code availability

The simulation code to reproduce the study presented herein is open source and available in the GitHub repository https://github.com/ludericv/information-market, and has been archived within the Zenodo repository: https://doi.org/10.5281/zenodo.8187233.
