# Supplementary material for: A blockchain-based information market to incentivise cooperation in swarms of self-interested robots
Source: Sci Rep. 2023 Nov 21;13:20417. doi: 10.1038/s41598-023-46238-1 (PMC10663462; doi:10.1038/s41598-023-46238-1)
Supplement: Supplementary file 1 — Supplementary Information. [file 41598_2023_46238_MOESM1_ESM.pdf]

Supplementary Material of the article: A blockchain-based information market to incentivise cooperation in swarms of self-interested robots

Ludéric Van Calck[1], Alexandre Pacheco[1], Volker Strobel[1], Marco Dorigo[1], and Andreagiovanni Reina[1,*]

[1]Institut de Recherches Interdisciplinaires et de Développements en Intelligence Artificielle (IRIDIA), Université Libre de Bruxelles, Brussels, Belgium

[*]andreagiovanni.reina@gmail.com

Movie S1 `https://youtu.be/havFlLrUpJY`
Movie S2 `https://youtu.be/YGlxwrH3_io`

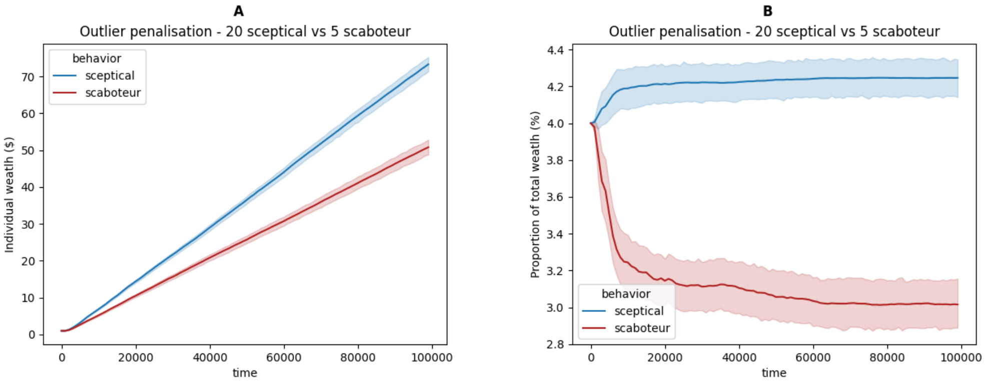

Figure S1: Average robot's wealth in a swarm of 20 sceptical and 5 scaboteur robots using the payment scheme of outlier penalisation (without staking) in 32 simulations. The transparent shades show the 95% confidence interval. (**A**) The absolute wealth of the scaboteurs increases at a slower rate than the one of honest sceptical robots; however, the wealth of both types of robots constantly increases. (**B**) The proportion of total wealth rapidly converges to a relatively stable situation where scaboteurs have a smaller proportion of wealth than honest robots; however, each scaboteur still collects about 3% of the total wealth. These results motivated the introduction of a new payment scheme, outlier penalisation with staking.

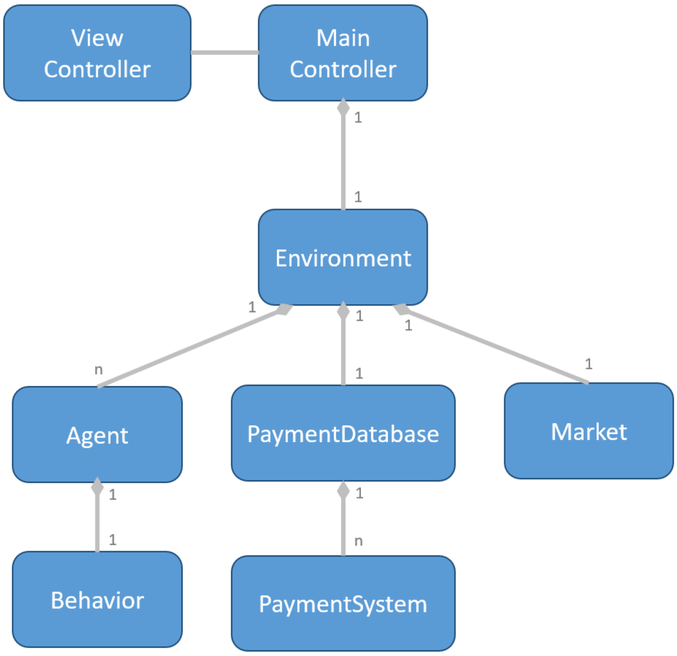

Figure S2: Simplified UML diagram describing the architecture of our multiagent simulator.

Text S1 Analysis of the methods to combine vectors of the navigation table

Choosing to buy more recent information relies on the assumption that information's age measures its accuracy. Because information is distorted through imprecise odometry estimates, older information is likely to have accumulated a larger error and to be less accurate. Even though this is true for information updated by a single robot, in the negotiation process, a robot needs to decide if newer information from *another* robot is indeed more accurate than its own. As a robot's intrinsic drift is a random variable (see Eq. (1) in the main text), newer information from a robot with a large drift may actually be less accurate than older information from a robot with a small drift. Therefore, there could be value in robots estimating their own drift, to more precisely assess the accuracy of their own information. To test whether it is worthwhile for robots to have an intrinsic estimate of their own drift, we conducted experiments where a target's age attribute was replaced with a *quality* attribute $0\% \leq q \leq 100\%$. At each timestep, the robots decrease this quality attribute (analogously to increasing their information's age attribute), and use it to determine whether or not they should buy another robot's target information. Since the quality attribute decreases over time, robots decide to buy information with a higher quality value than their own (instead of buying information with a lower age value than their own).
We tested three different ways to decay the quality attribute.

- *constant decay*: the robot decreases the quality attribute of each piece of information in its navigation table by a constant c_1 at each step of the simulation:

$$q_{t+1} = q_t - c_1. \tag{S1}$$

  This is exactly analogous to using an age attribute and increasing it by 1 at each step. This method does not take into account a robot's intrinsic drift.

- *linear decay*: the robot decreases the quality attribute of its information by an amount proportional to its intrinsic drift (we assume robots have perfect knowledge of their drift bias μ even though they would need to estimate it in practice):

$$q_{t+1} = q_t - c_2\,|\mu|. \tag{S2}$$

- *exponential decay*: Finally, we test a third method where the quality of information decreases exponentially with a robot's drift at each time step, which penalises heavy drifters' information even further.

$$q_{t+1} = q_t\, c_3\,|\mu|. \tag{S3}$$

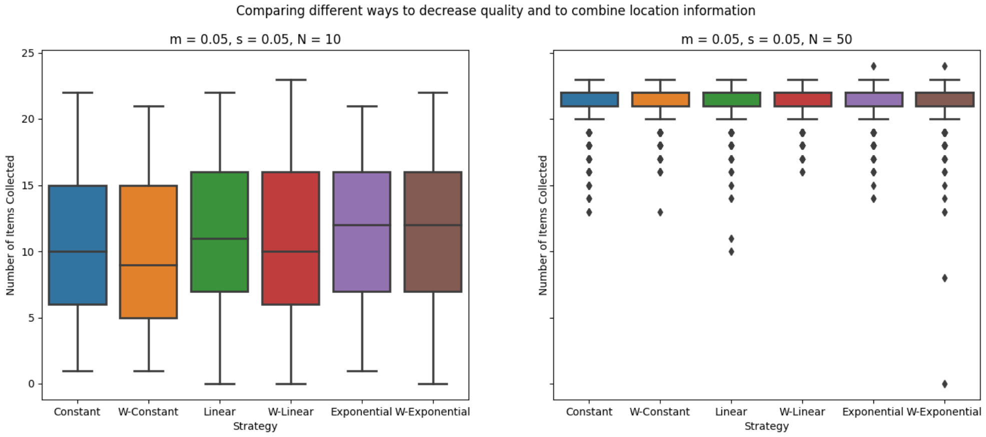

Figure S3: Comparison of different information quality and combination strategies for varying population sizes.

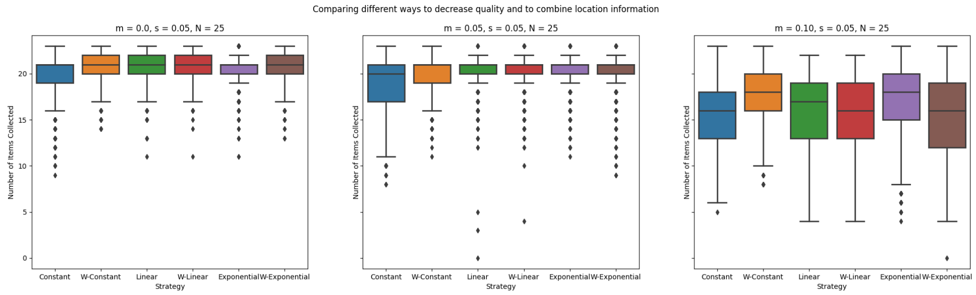

Figure S4: Comparison of different information quality and combination strategies for varying drift sampling parameter m_μ.

For these equations to make sense, the parameters c_1, c_2, c_3 must be positive (i.e. $c_1, c_2, c_3 > 0$) and $c_3 * |\mu| < 1$.

Furthermore, we distinguish two different methods a robot can use information it buys from another robot. The first method consists of directly replacing the previous piece of information with the new bought information for the same site type (i.e. food or nest) in the buyer's navigation table. The second method consists of combining the new information with the previous information using a weighted average to form a new entry in the buyer's navigation table replacing the previous piece of information (similarly to Eq. (4) and Eq. (5) in the main text). Since we are using quality attributes instead of age, the equations are:

$$\mathbf{x} = \frac{q_{buyer}}{q_{buyer} + q_{seller}} \cdot \mathbf{x}_{buyer} + \frac{q_{seller}}{q_{buyer} + q_{seller}} \cdot \mathbf{x}_{seller}\,; \tag{S4}$$

$$q = \frac{q_{buyer} + q_{seller}}{2}\,, \tag{S5}$$

with q representing the information's quality attribute and $\mathbf{x}$ the 2D relative position vector.

Three quality decay methods (constant, linear, and exponential) and two information combination methods (direct replacement and weighted average) sum up to six total combinations to test, to ultimately decide the best overall information accuracy measurement and combination strategy. We compare the six strategies by running 64 simulations per condition and measuring the swarm performance as the number of items collected by each robot. We ran experiments with swarms composed of naive robots where we varied the population size (N) and the drift parameters m_μ and s_μ, of Eq. (1) in the main text. The results of these experiments are reported in Figures S3, S4 and S5. In these Figures, we label the six strategies with the decay method name (constant, linear, exponential) with the letter "W" as prefix in the case of the weighted average combination, and with no prefix for the direct replacement method. For example, "W-Linear" indicates the results for the strategy with weighted average information combination and linear quality decay, whereas "Constant" refers to the strategy with direct information replacement and constant quality decay.

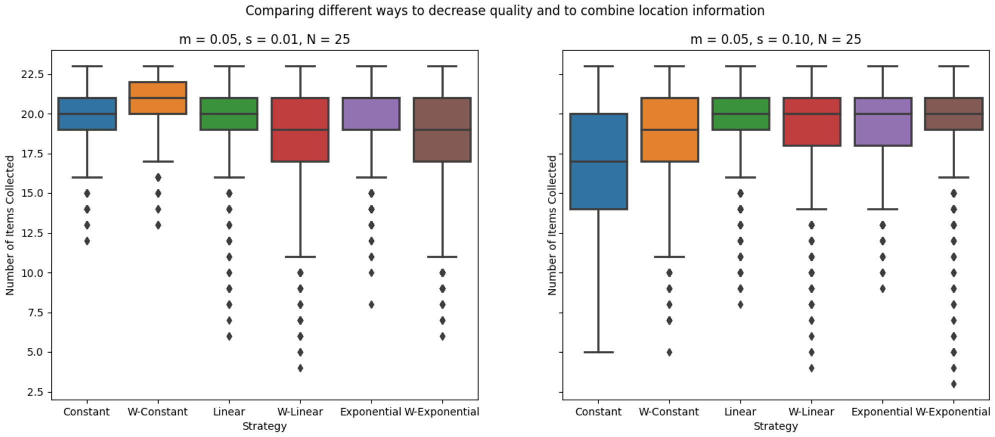

Figure S5: Comparison of different information quality and combination strategies for varying drift sampling parameter s_μ.

Since the swarm performance (in terms of number of items collected) is similar between all six strategies for all experiments, we conclude that estimating a robot's intrinsic drift, which is necessary to use the quality measure used for linear and exponential quality decay, (which would not be as accurate as for these control experiments where we gave perfect information about the magnitude of their drifts to the robots) would not be worth it compared to simply using a constant decay. Furthermore, since the weighted average information combination seems to produce a slight increase in performance compared to information replacement for a constant quality decay, and it can be implemented with close to no additional computational cost, we decide to use it. Finally, since an abstract decreasing quality attribute is completely equivalent to a more concrete increasing age attribute, we use an age attribute instead of a quality attribute in our other simulations for this paper.

Text S2 Blockchain smart contract in Solidity

```
// SPDX-License-Identifier: GPL-3.0

pragma solidity >=0.7.0 <0.9.0;

/**
 * @title Information Market
 * @dev An information market for social navigation in robot swarms
 */

contract InformationMarket {

    constructor() payable {}

    // Structure that contains the information sold by the selling robot
    struct Information {

        uint8 target; // food site or nest site?
        uint256 age; // age of the information
        uint256 maxBlockNumber; // transaction needs to be mined before this block
        uint256 x; // x position to the target in the buyer's coordinates
        uint256 y; // y position to the target in the buyer's coordinates
        address payable informationSeller; // public address of the seller
    }

    // Total number of collected items
    uint256 public totalItems;

    // Mapping from buyer robots to all contributors
    mapping (address => Information []) public contributors;


    /**
     * @dev Verify that the buyer's signature of the light contract is valid
     * @param target nest site (1) or food site (2)?
     * @param age of the information
     * @param maxBlockNumber timestamp before this transaction must be mined
     * @param buyer of the information
     * @param signatureBuyer of the buyer of the information
     */
    function verifySignature(uint8 target, uint256 age, uint256 maxBlockNumber,
                             address buyer, bytes memory signatureBuyer)
        public pure returns (bool) {

        bytes32 messageHash = getMessageHash(target, age, maxBlockNumber);
        address recoveredBuyer = recover(messageHash, signatureBuyer);

        return buyer == recoveredBuyer;
    }

   /**
     * @dev Hash the light contract
     */
    function getMessageHash(uint8 target, uint256 age, uint256 maxBlockNumber)
    internal pure returns (bytes32) {
        return keccak256(abi.encodePacked(
            target,
            age,
            maxBlockNumber
        ));
    }

    /**
```

```
 * @dev Recover signer based on messageHash and signature
 * @param messageHash hashed message
 * @param signature of the message
 * @return address of the signer
 */
function recover(bytes32 messageHash, bytes memory signature)
internal pure returns (address)
{
    (bytes32 r, bytes32 s, uint8 v) = split(signature);
    return ecrecover(messageHash, v, r, s);
}

/**
 * @dev Split signature into r, s, and v (required for ecrecover)
 * @param signature of the message
 * Function based on Smart Contract Programmer's video "Verify Signature"
 * https://youtu.be/vYwYe-Gv_XI (accessed July 10, 2022)
 */
function split(bytes memory signature) internal pure returns
(bytes32 r, bytes32 s, uint8 v)
{
    require(signature.length == 65, "invalid signature length");

    assembly {
        r := mload(add(signature, 32))
        s := mload(add(signature, 64))
        v := byte(0, mload(add(signature, 96)))
    }
}

/**
 * @dev Reveal relative position to the target
 * @param target nest site (1) or food site (2)?
 * @param age of the information
 * @param maxBlockNumber timestamp before this transaction must be mined
 * @param x position to the target in the buyer's coordinates
 * @param y position to the target in the buyer's coordinates
 * @param informationBuyer who signed the contract
 * @param signatureBuyer signature of the buyer of the information
 */
function revealInformation(uint8 target, uint256 age, uint256 maxBlockNumber,
    uint256 x, uint256 y,
    address informationBuyer, bytes memory signatureBuyer) public
    {

    // Perform the three security checks

    // 1. Verify that the seller is not too late
    // (i.e. max block number in the light contract
    // is smaller than or equal to the current block number of blockchain)
    require(block.number <= maxBlockNumber,
            "maxBlockNumber: Tx was mined too late");

    // 2. Verify signature of the buyer
    require(verifySignature(target, age, maxBlockNumber,
            informationBuyer, signatureBuyer),
            "Signature is invalid");

    // 3. Verify that the information is unique
    require(isInformationUnique(target, age, maxBlockNumber,
            msg.sender, informationBuyer),
            "Information is not unique");
```

```
        // Create information structure and push it to the buyer's list
        Information memory information;

        information.target = target;
        information.age = age;
        information.maxBlockNumber = maxBlockNumber;
        information.x = x;
        information.y = y;
        information.informationSeller = payable(msg.sender);

        // Add this selling robot to the list of contributors
        contributors[informationBuyer].push(information);
    }

    function isInformationUnique(uint8 target, uint256 age, uint256 maxBlockNumber,
                        address informationSeller, address informationBuyer)
         internal view returns (bool)
    {

        // Iterate over contributors list and check if information is already stored
        for (uint256 i = 0; i < contributors[informationBuyer].length; ++i) {

            Information memory currentInfo = contributors[informationBuyer][i];
            if (currentInfo.informationSeller == informationSeller &&
                currentInfo.target == target &&
                currentInfo.age == age &&
                currentInfo.maxBlockNumber == maxBlockNumber) {
                    return false;
                }
        }
        return true;
    }

    /**
     * @dev Buy an item from a robot
     * @param itemSeller address to buy from
     */
    function buyItem(address payable itemSeller) public payable {

        // Transfer 5 ether to the item seller
        itemSeller.transfer(5 ether);

        // Compute payouts for the contributors (simplified)
        for (uint256 i = 0; i < contributors[itemSeller].length; ++i) {
            contributors[itemSeller][i].informationSeller.transfer(1 ether);
        }

        // Reset the information list for this robot
        delete contributors[itemSeller];
    }
}
```
